# Piezoelectric Energy Harvesting towards Self-Powered Internet of Things (IoT) Sensors in Smart Cities

**DOI:** 10.3390/s21248332

**Published:** 2021-12-13

**Authors:** Iman Izadgoshasb

**Affiliations:** Faculty of Science and Engineering, Southern Cross University, Gold Coast, QLD 4225, Australia; iman.iz@gmail.com

**Keywords:** piezoelectric, energy harvesting, smart cities, Internet of Things

## Abstract

Information and communication technologies (ICT) are major features of smart cities. Smart sensing devices will benefit from 5 G and the Internet of Things, which will enable them to communicate in a safe and timely manner. However, the need for sustainable power sources and self-powered active sensing devices will continue to be a major issue in this sector. Since their discovery, piezoelectric energy harvesters have demonstrated a significant ability to power wireless sensor nodes, and their application in a wide range of systems, including intelligent transportation, smart healthcare, human-machine interfaces, and security systems, has been systematically investigated. Piezoelectric energy-harvesting systems are promising candidates not only for sustainably powering wireless sensor nodes but also for the development of intelligent and active self-powered sensors with a wide range of applications. In this paper, the various applications of piezoelectric energy harvesters in powering Internet of Things sensors and devices in smart cities are discussed and reviewed.

## 1. Introduction

**Figure 1:**
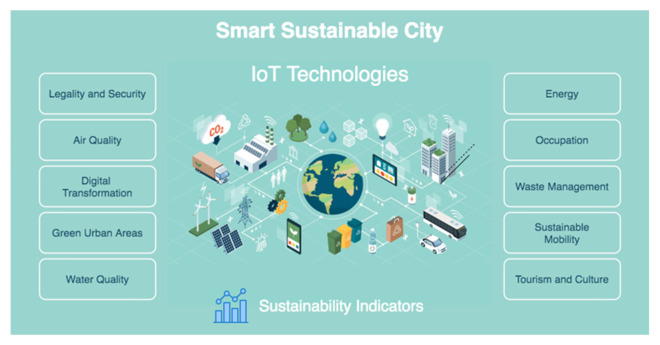
Major areas in smart cities that use IoT technologies, adopted from [2].

Smart cities play an important role as innovation drivers for businesses in a variety of industries, including health, the environment, and information and communication technology (ICT). The future smart city leverages smart innovation ecosystems to improve the overall quality of life of citizens [1]. Smart cities aspire to turn rural and urban regions into democratic innovation hubs [2,3], with innovation ecosystems boosting the collective intelligence and co-creation capacities of user-citizen communities to develop creative living and working spaces. As shown in Figure 1, smart cities are pioneering open, user-driven innovation in order to demonstrate the benefits of future internet-enabling services. Smart cities could be established and developed using advanced ICT infrastructure and Internet of Things (IoT) devices. The IoT has been identified as a determining pillar of ICT, characterised as a huge network architecture built on the capacity to share information and connect it with physical items, such as electric automobiles or drones [4] and virtual “things” [5]. The IoT is comprised of digital technologies, semantic languages, and virtual identities [6]. The IoT enhances the reliability and effectiveness in operation and administration of smart ecosystems [7]. For example, the installation of smart sensors in San Francisco Park allowed customers to get instant information while waiting for a parking spot, reducing wait times [8]. Smart sensors collect data that can be transferred to devices through a wireless link and proprietary software. A smart grid capable of handling fluctuating power supplies will be necessary for the future sustainable cities. One of the major aims is to use local renewable energy sources as much as is feasible [9,10,11]. Solar panels and batteries can be deployed on a larger scale in cities as part of such a system. They will be able to communicate with the grid and provide and store electricity in accordance with changes in demand. In addition, small-scale energy-harvesting systems, such as vibration-based harvesters, have attracted the attention of many researchers over the last decades [12,13,14].

Given the complexity of energy systems of urban centres, one of the most pressing processing concerns is energy management [12,13,14]. Smart cities aim to enhance the quality of life of citizens by utilising technology for the management of the available resources [15]. This primary role in smart cities is played by sophisticated and progressive systems that automate and improve processes within the cities. These systems can use intelligent devices that can monitor and control the infrastructure of cities autonomously. The past decade has seen the implementation of numerous applications, as well as the resolution of challenges linked with these applications [16]. Renewable-energy harvesters are among the applications that have been deployed in such cities, having less negative impact on the surrounding environment. Researchers have developed a variety of methods for harvesting energy from diverse sources, including wind, solar, heat, motion, and vibration. Scholars have conducted many studies on the transition of kinetic energy from surrounding energy sources, such as humans [17], wind or airflow [18], and water current or waves [19].

This review provides an overview of the protentional applications of vibration-based energy harvesters that have the capability of generating electricity for remote sensors and devices in smart cities.

## 2. Piezoelectric Energy Harvesting: Concepts and Methodologies

Researchers have used three harvesting mechanisms to generate electricity from vibration sources consisting of electromagnetic, electrostatic, and piezoelectric energy harvesting. Piezoelectric energy harvesters are promising candidates to generate power from surrounding vibration sources. They have the advantage of converting mechanical strain to electrical energy without consuming any additional power and have an enormous power density, ease of application, and the possibility of fabrication at different scales. Piezoelectric energy-harvesting systems can produce maximum power output if their resonant frequencies match the excitation vibration frequencies. This electrical power can be consumed by microelectronic devices or can be accumulated in batteries. The vibration to electrical energy conversion method was first introduced by Williams and Yates back in 1996 [20] but has received significant attention in recent years. They proposed a lumped parameter second-order system that shows the relationship between an input vibration, y(t), and the output relative displacement, z(t). The dynamic equation of the proposed system can be shown as:

(1) $$m\ddot{z}+d\dot{z}+kz=-m\ddot{y}$$

where $\ddot{y}\left(t\right)$ is the input acceleration, z(t)  is the displacement of the tip mass, *k* is the spring constant, *d* is the damping ratio, and *m* is the mass of the cantilever beam. G(s) or transfer function then can be given as:

(2) $$G\left(s\right)=\frac{-m}{ms^{2}+ds+k}$$

The natural frequency of the system, $\omega_{n}$, can then be obtained:

(3) $$\omega_{n}=\sqrt{\frac{k}{m}}$$

**Figure 2:**
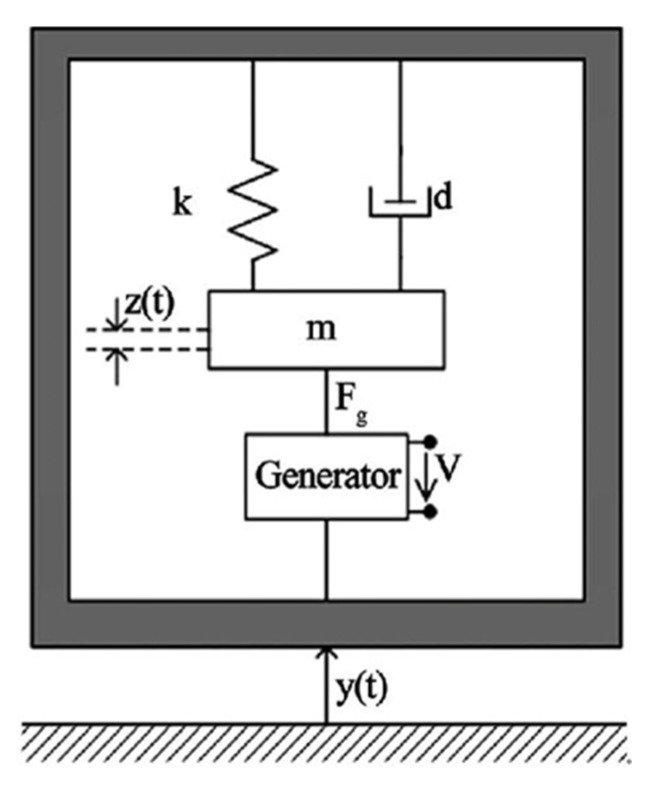
The dynamic model of a vibration-based energy harvester.

It is evident that the spring and the mass are two effective parameters controlling the resonant frequencies. For the cantilever beam, which is shown in Figure 2, the resonant frequency of the system is given by Equation (4), where Y is Young’s modulus of the beam material; *l*, *h*, and *w* are the length, thickness, and width of the cantilever beam, respectively; *m* is the tip mass; and $m_{c}$ is the mass of the beam.

(4) $$f_{r}=\frac{1}{2\pi }\sqrt{\frac{ywh^{3}}{4l^{3}\left(m+0.24m_{c}\right)}}$$

The resonant frequency tuning methods are categorised into two modes: active or passive. If the tuning mechanism operates periodically and consumes energy only during the tunning operation, it can be called the “passive” method. In contrast, “active” tuning is described as a tuning mechanism that is constantly applied to the system, regardless of the current operating frequency of the system.

**Figure 3:**
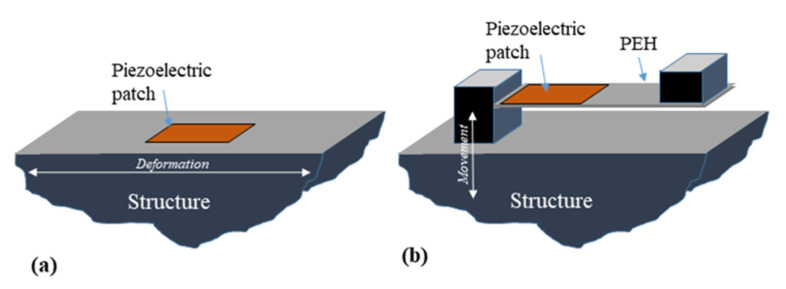
Piezoelectricity energy harvesting systems (**a**) bonded piezoelectric material (**b**) piezoelectric cantilever beam attached to the system.

The piezoelectric effect is a material feature that converts mechanical energy in the form of pressure or vibration into electrical energy [21]. Stress can cause ions to reposition, generating electrical displacements, which can result in spontaneous polarisation and the formation of an electric field. Ferroelectrics are a kind of substance that have the ability to conduct electricity. Energy harvesters based on piezoelectric technology have been deployed to collect energy from a variety of vibrations. There are two installation schemes to harvest energy from piezoelectric materials: (a) directly installing the piezoelectric element on the host structure undergoing surface deformation and (b) attaching cantilever beams with piezoelectric element bonded on a host structure where large base excitation is expected. Both schemes are schematically shown in Figure 3. For the first scheme, the host structure’s dynamic is not changed during the process of power harvesting. Thus, there is limited room for improvement in the energy-harvesting system. For the second scheme, the cantilever beam is attached to the base structure, and enhancement of efficiency is possible by tuning the dynamic of the harvester. The second design is often preferred if the system has the capability of hosting the transducer because a certain model of piezoelectric energy-harvesting systems can be developed.

In reality, some researchers have made an effort to attach piezoelectric materials by attaching them directly to human skin or wearable clothes (installation scheme, Figure 3a), while many others have tried to make an energy-harvesting device that can generate vibration based on various human body motions (installation scheme, Figure 3b).

**Figure 4:**
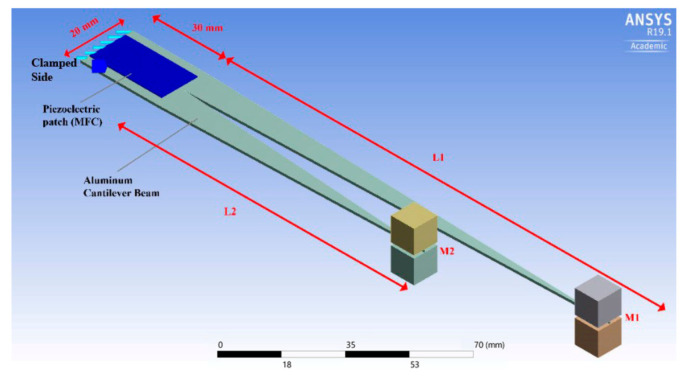
Schematic of a multi-resonant piezoelectric energy harvester design adopted from [28].

Yang et al. [22] evaluated piezoelectric applications, with a particular emphasis on approaches that provide high-power outputs while maintaining a broad operating bandwidth. With the help of theoretical calculations and experimental tests, Yang et al. [23] were able to demonstrate the performance of piezoelectric energy-harvesting systems from linear and non-linear vibration sources. They showed that the output power was proportional to the excitation and response phase differences. Wei and Jing [24] evaluated the model of piezoelectric vibration-based energy harvesters, in which ceramics or polymers were used as base materials; they discussed the implications of this work. Zhang et al. [25] concentrated on the non-linear resonance frequencies and widening the bandwidth of piezoelectric energy harvesters, as well as analysing the non-linear effect in the vibration systems. Moreover, Yildirim et al. [26] presented a technique for enhancing the conversion of ambient energy, as well as tuning the resonance frequency of the system. Matching frequency is necessary to enhance the performance of energy-harvesting systems. With the help of electrical characteristics measured under various bending conditions, Cao et al. [27] demonstrated the use of the transverse piezoelectric effect to create a water-resistant and self-powered sensor. The results show that the electrical characteristics have a linear dependence on voltage and an inverse-square-root dependence on power. Izadgoshasb et al. [28] introduced a new geometry for the cantilever beam that can widen the frequency bandwidth. As shown in Figure 4, they used a branched beam in their design, which resulted in obtaining two close resonant frequencies.

To depict the energy-conversion process, Cao et al. [29] undertook experiments to determine the basic principle of dipole movements in polymer-based piezoelectric materials. The internal impedance of instruments, as well as the sampling rate of the devices, had a substantial impact on the energy-transmission process. Various studies have been carried out over the last decade to boost output power and improve the performance of harvesting equipment. In order to create piezoelectric energy harvesters, three major strategies have been employed. The first was selecting a suitable operating mode, the second was modifying materials, and the last was altering the structure of the harvester itself. There are two different operating modes for piezoelectric harvesters, which are known as mode 33 and mode 31. Researchers [30] investigated the differences between two operating modes of a cantilever-based piezoelectric. They discovered that the mode 31 devices had a larger output power and open-circuit mean voltage than the mode-33 devices but had a lower resonance frequency and maximum open-circuit voltage when compared to the mode 33 device [30]. Additionally, Kim et al. [31] investigated the influence of both modes on the performance of an energy harvesting system and developed the Norton equivalent representation for each mode. These researchers came to the conclusion that mode 33 has the potential to provide only a moderate improvement over mode 31.

The presence of water vapour and oxygen in the surrounding air might have an impact on piezoelectric materials and their performance. To overcome these issues, a variety of materials have been tested to structure piezoelectric harvesters, including conventional ceramic materials, polymers, and aluminium nitride, among others. Other researchers [32,33] employed lead-free piezoelectric sheets to create MEMS harvesters for their experiments. The structure of the piezoelectric harvester is critical in terms of increasing the harvester’s output power and efficiency. Various architectures have been designed and explored in order to improve the performance of harvesters.

The cantilever harvester is the most common form of piezoelectric harvester seen in the field. An array of cantilevers has been created in order to provide flexibility and high efficiency in energy harvesting. The thickness of the supporting layer and the alignment of different layers in arrayed harvesters have a significant impact on the voltage, current, and output power, and these factors must be taken into consideration when designing a harvester. Arrayed harvesters were shown to react at lower frequencies than single-cantilever harvesters [34,35]. Ring form [36,37], cylinder shape [38,39], and sandwich structure [39], which was made of two piezoelectric sheets with a metal shim sandwiched between them, are all examples of structures that have been created specifically for the purpose of enhancing piezoelectric energy-harvesting systems.

Researchers also performed a study on the generation of electricity from rain drops [40]. The harvester was constructed from lead zirconate titanate films in two layers and a sandwiched shim layer. Droplets that landed on the cantilever’s top would cause the cantilever to vibrate as a result of the kinetic energy transferred from them. The electromechanical connections were used to compute the amount of output energy created as a result of this influence. The performance of the energy harvester was altered by tuning the resonant frequency since the amount of harvested power rises as the resonant frequency decreases. Lower resonance frequencies result in more output power. Using experimental data, it was discovered that the power yield was 37 times more than that obtained in prior studies. Later in the same year, a more advanced version of this harvester for similar applications was introduced. They increased the number of layers to five, all of which were constructed of the same material, lead zirconate titanate. They claimed that the new version of energy harvester was able to generate an output power of 400 mJ. Researchers have found that the use of many layers of PZT cantilevers can increases the output-power yield. The strength and position of the drops also have an impact on the harvester’s output yield. There were three different rain intensities (moderate, low, and intense modes) investigated in previous studies [41]. Similarly, a study was conducted to investigate the amount of strain in a cantilever beam as a result of a radio frequency (RF) propagation signal being broadcast. A cantilever was actuated when the applied RF propagation field meets the resonance frequency of the structure. Lead-zirconate-titanate was a base material of the cantilever and measured 7 mm in length and 1 mm in width and had a thickness of 0.24. This electrode had gold-plated electrodes, with a total thickness of 80 nm [42]. A large number of researchers have shown an interest in improving energy harvesters. This served as the impetus for the development of a novel approach that supports searchers in optimising energy harvesters in a simple and efficient manner. It is suggested that the electrical output power of a standard cantilever can be estimated using an approach that is based on the analogy of electromechanical input impedance. The models are derived based on power-system theories, electromagnetic theories, as well as direct mechanical-to-electrical comparison. These models calculate output power based on the harvester’s material type, as well as its dimensions. After experimentally establishing the concept, it was determined that the model can provide critical extra information for improving harvester operation and design when compared to traditional methods [43]. The harvester design necessitates a power conversion circuit, known as a DC/DC converter [44].

The innovative concept is to employ a bias-free device capable of producing enough power for battery-free sensor operation for distant applications. The harvester can be made up of a self-biased oscillator (a piezoelectric vibrational oscillator), which controls the frequency and voltage of the switches in the DC/DC converters, allowing the output DC voltage to be controlled and power to be saved without the use of an external biassing source. A piezoelectric energy harvester’s structure consists of a piezoelectric sheet or a beam connected to a vibrating mechanical frame. This piezoelectric layer bends as a result of the vibration, and the bending generates electricity. It is common to use a proof mass at the free end of the cantilever beam to increase the beam’s bending, which can result increased output power. This proof mass can be changed to tune the structure’s resonance frequency, making it closer to that of the vibration source, optimising output power as much as feasible. Current research is actively pursuing piezoelectric harvester modelling to fulfill the needs of design and development of these harvesters [45,46,47]. It is also desirable to have a complete model that efficiently ties the amount of harvested power to the structure of piezoelectric energy-harvesting systems and the base material. The energy-harvesting component of the piezoelectric harvester circuit is divided into two sections. The generated voltage from the piezoelectric patch is routed to an AC/DC full-wave bridge rectifier that converts the AC voltage into DC [48]. In the next step, the converted DC voltage would be enhanced using a DC/DC booster that then can be transferred into the storage component and finally to low-power applications that do not need batteries. The voltage outputs of the piezoelectric cantilever are transformed to direct-current voltages by means of two independent full-bridge rectifiers. In this paper, the feasibility of electric power generation using piezoelectric materials on a larger scale is investigated. It is necessary to take into account the link between piezoelectric materials, the energy conversion process, working in a natural frequency range, and mechanical characteristics to produce the highest output power. Creating power through vibration necessitates the matching of the frequency of the vibration source with the natural frequency of the piezoelectric energy harvester. However, the harvesters reviewed in this paper are mostly non-resonant energy-harvesting systems. The geometry of piezoelectric energy harvesters and their resonance frequencies can affect the power output of the systems. Table 1 compares the power output and voltage of several piezoelectric energy-harvesting systems.

## 3. Smart City Piezoelectric Applications

There are several sources of vibration and motion in smart cities that could be used for the purpose of energy harvesting. The kinetic energy from human movements, wind and air pressure, vehicles, ocean waves and water pressure, buildings and structures, and bridges and roads can be directly converted to electrical energy with the help of piezoelectric energy-harvesting systems. In this section, various studies of the above-mentioned sources will be reviewed and discussed.

**Figure 5:**
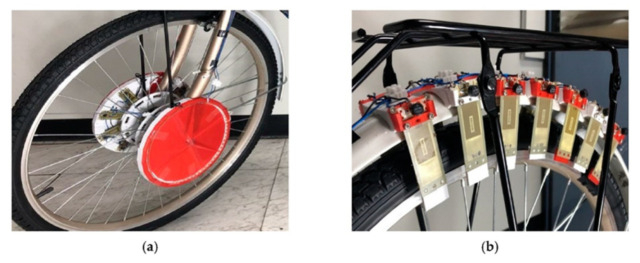
The installation of piezoelectric energy harvesters on a bicycle. (**a**) Piezoelectric harvesters installation distance at front wheel; (**b**) Piezoelectric harvesters installation distance at rear wheel fenders., adopted from [1].

Human movements can be considered as one of the protentional sources of energy in smart cities. Ali et al. [56] studied the use of piezoelectric energy-harvesting devices in biomedical applications. The mechanical energy that is produced by human beings, such as by muscular relaxation, bodily movement, blood circulation, lung expansion, and cardiac motion, could be converted into electricity using their designs. These researchers claimed in some cases, it is possible to harvest a maximum voltage of 10 V and a power density of 0.27 μW/cm^2^. Piezoelectric harvesters have the potential to be employed in wearables, such as shoes, clothing, and watches [57]. A number of harvesters have been built to produce electrical power from arm motions since 1990; a good example of this is the Maestro brand of Swiss timepieces, which has been in existence since 1890. The kinetic energy from movement mechanisms can be converted to electrical energy and can be stored by the wristwatch’s embedded micro-generators. Walking is one of the most common actions performed by humans due to generating a large displacement. Piezoelectric materials have been developed to harness the energy created by walking by placing them into shoes to harvest energy from human weight or by attaching the energy harvester to the leg/foot so it can be excited by a walking/swing motion. Generated energy might be used in the future to charge mobile phones and health devices. The double-pendulum system in one example is adopted to improve the performance of energy harvesting from human walking motions [58]. Kymissis and colleagues [59] conducted a test on an energy harvester that was embedded in a shoe and contained three harvesters. In other case, a wearable device is used to harvest energy from a backpack by substituting the bag’s strap with a PVDF strap [60]. The electrodes on the surface of the strap were constructed using an electrostatic self-assembly method, which ensured that the strap could withstand heavy loads. The results showed that this device was capable of harvesting around 45.6 mW. A staircase was created in another instance to gather energy from human movements [61]. The excitation signals were dependent on the walking environment, as well as the individual’s features [62]. Jettanasen et al. [1] developed a piezoelectric energy harvester to scavenge energy from bicycles while riding them in smart cities. Figure 5 shows the structure of their work. Li and Strezof [63] investigated the possibility of scavenging human walking energy in Macquarie University library. They focused on the areas of the building with the greatest levels of motion. They discovered that the greatest locations for power generation were the cafeteria, the loopy meeting rooms, and the main entrance. Pavegen tiles were utilised to capture energy from movement along the walkway. The number of tiles was determined by their sizes and deployment technique. The ideal deployment strategy was chosen based on the breadth and length of the routes, since the tiles were arranged in both lengthwise and width wise modes [63].

**Figure 6:**
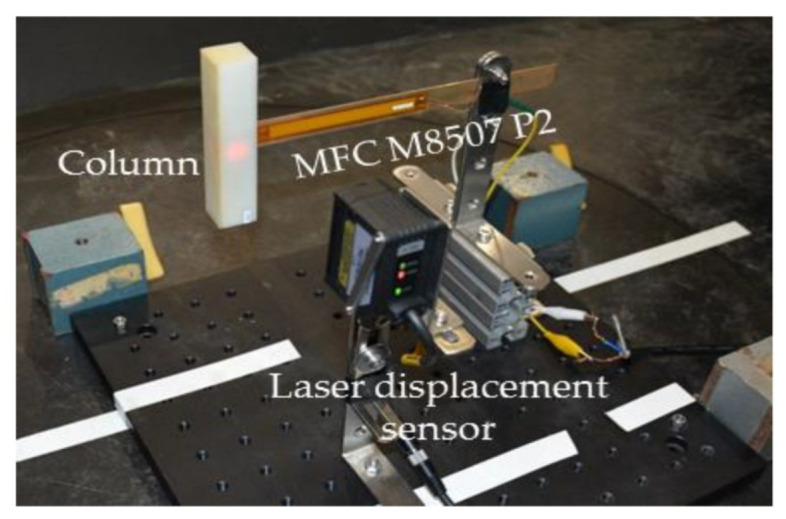
Galloping piezoelectric energy harvester with V-shaped groove in low wind speed, adopted from [66].

Additionally, it is possible to use the kinetic energy from fluids to generate electricity in smart cities. Piezoelectric energy harvesters are able to be placed in water to harness the energy generated by the longitudinal motion of sea waves. Researchers studied an energy harvester that was made up of a cantilever beam with attached piezoelectric patches [64]. They used a proof mass to decrease the natural frequency of the proposed energy harvester. The output power was determined to be proportional to the beam thickness and its dimensions, the used mass at the free end of cantilever, wave height, water depth, as well as the ratio of water depth to wave height [64]. In another case, a windmill prototype with 10 piezoelectric harvesters was placed in a circular pattern and was tested in winds ranging from 1 to 12 mph. When the wind speed hit 10 mph, the harvester’s output power was around 7.5 mW [65]. Zhao et al. [66] introduced the galloping V-shaped piezoelectric wind-energy harvester, which was able to produce maximum of 1 mW at a flow velocity of 10 m/s, which is close to the wind speed in urban areas. Their design is shown in Figure 6. A pressured water current was employed as an energy source, which was then converted to electrical energy by using a shear-mode harvester [67]. Spornraf et al. [68] demonstrated piezoelectric bend transducers triggered by laminar flow. In another application, piezoelectric harvesters have been utilised to power the electronic systems of aeroplanes. The harvester transforms kinetic energy from the intake air current to electricity during flights. A device was constructed and examined by simulating airflow in the air cylinders. These findings indicated that the airflow velocity, sound pressure, and open-circuit voltage were linearly related. This demonstrates the suitability of harvesters for aeroplane applications [69].

The generated vibrations from vehicle movements in smart cities can also be used as a source of energy. A piezoelectric harvester was positioned on a vehicle damper to harvest electrical energy from tyre movements [70]. Energy harvesting has been accomplished via the utilisation of pneumatic tyre deflections under borne load. This energy is dependent on the geometry of the tyres, the vehicle’s speed, and the air pressure in the tyres. Piezoelectric stacks are integrated into the tyre and are made of lead zirconate titanate. In this study, the harvester is represented by the first-mode vibration of a cantilever. Despite the fact that it captures only modest quantities of energy, it is sufficient to power wireless sensors [71]. Kulkarni et al. [72] were the first to demonstrate the use of piezoelectric energy harvesters in automotive systems; they demonstrated that a gasoline injector that uses piezoelectric materials can be more precise than a traditional one.

**Figure 7:**
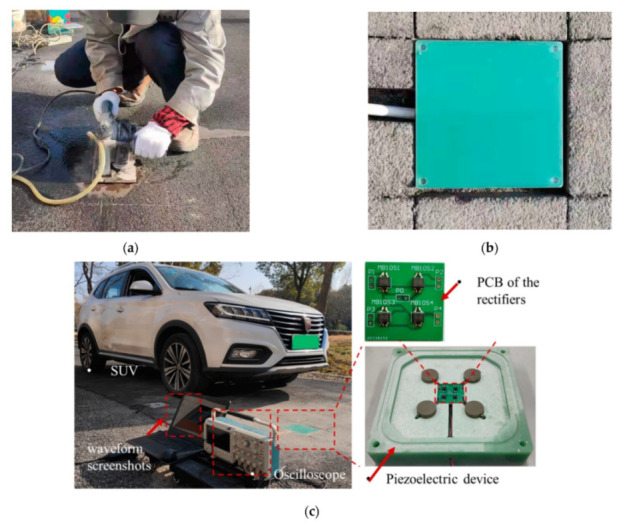
On-site tests of the piezoelectric device: (**a**) cutting the pavement surface, (**b**) installing the PEH, and (**c**) applying vehicle load. Adopted from [78].

Infrastructure in smart cities, such as roads, buildings, and bridges, can be also considered as sources of vibration. To exploit the vibrations of high-rise structures caused by wind, Xie et al. [73] constructed a linked piezoelectric cantilever with a proof mass. They showed that the device can be optimised by examining the influence of the connected mass position, length, radius, and the ratio of the piezoelectric component to the beam. They refined their design and exhibited a unique harvester. The new version was made up of two sets of generators that were linked in series through a shared shaft. This harvester was able to generate power from harmonic motions and, at the same time, acted as a damper, dissipating structure vibrations [74]. In similar research, Chen et al. [75] looked at the use of piezoelectric materials in the structure of buildings, as well as the use of these materials in the bodies of energy harvesters, actuators, and sensors. Elhalwagy et al. [76] provided a feasibility study on the use of piezoelectric energy harvesters in building floors and also adopted a strategy for maximising the amount of energy produced by these harvesters. According to Garimella et al. [77], a system for producing electrical power from vibration has been developed, with the capability of generating energy from undesirable vibrations. Li et al. [78] recently developed a piezoelectric energy harvester that can generate electricity from roadways while cars are passing. As shown in Figure 7, they designed a device that can be placed in pavement. They were able to harvest a maximum voltage of 170 V and a maximum power of 92 mW.

Moure et al. [79] examined the integration of piezoelectric cymbal harvesters in 29-cm-diameter asphalt for the first time. It was discovered that each cymbal collects up to 16 μW of electricity from the passage of a single large car. When 30,000 cymbals were integrated into a 100 m stretch of road, the generated energy was between 40 and 50 MW h/m^2^, which equates to around 65 MW per year. The results show that traffic on bridges can generate enough vibration to be used as a source of kinetic energy. One study revealed that such devices are capable of harvesting up to 12.5 mW of energy. Experiments were undertaken to show the operation of wireless systems utilising accumulated energy harvested from vibration of a rural highway bridge with low traffic flow [80]. Road-energy harvesting has also been studied by Xu et al. [81], who investigated the electromechanical conversion properties of piezoelectric materials applied to pavement and demonstrated that it is viable.

In another case, piezoelectric energy harvesters were used to power the transmitters tracking insect movements. Shearwood et al. [82] used a small piezoelectric energy harvester, a battery-less 5.8 GHz transmitter, and a compact antenna on 90 mg honeybees. The harvester was able to generate more than 2 V, which was enough to power the transmitter. In similar research work, Aktakka et al. [83] designed and fabricated a small piezoelectric energy harvester that can harvest energy from the wing motion of green June beetles. Their microgenerator was a non-resonant piezoelectric bimorph operating in d31 mode. They claimed that a maximum power of 115 μW was able to be generated from insect movement. They also found that a direct connection between the flight muscles of the insects and the microgenerator can result in increased power output. Using piezoelectric energy harvesters, it might be possible to monitor the movements of insects in future smart cities.

## 4. Conclusions

Ambient energy-harvesting sources in smart cities have sparked considerable interest because they can provide power for small micro-electronic devices and real-time IoT sensors. Piezoelectric materials are great candidates for this purpose. In this review paper, current piezoelectric energy-harvesting systems for kinetic energy sources were discussed and reviewed. Research with a focus on piezoelectric energy harvesting from various energy sources, such as wind, ocean, vehicles, roads, bridges, infrastructures, and human and animal movements, were explained to verify the potential of using mechanical energy harvesters to power advanced IoT applications in smart cities. The operational parameters and mechanical architectures of various energy harvesters were elucidated. Optimized construction, proper materials, and power management circuits are all scientifically proven to increase output power.

There have been significant recent successes concerning supporting services in smart cities by using IoT sensors and applications that could be powered by improved piezoelectric energy harvesters.

## Figures and Tables

**Table 1:** Various Piezoelectric energy-harvesting systems and their power output, extracted from [49].

Description of Piezoelectric Energy-Harvesting System	Design Geometry/Dimension	Resonant Frequency	Power Output/Voltage	Ref.
PZT and AIN device	Piezoelectric patch was placed on the top of the beam and was sandwiched between two electrodes	300, 700 and 1000 Hz	1–100 μW	[50]
PZT cantilever beam	Dimension: 13.5 mm × 9 mm × 192 μm	13.9, 21.9 and 48.5 kHZ	2.4 V with 5.2 MΩ load, 1.01 μW	[51]
PZT cantilever beam with interdigital electrodes	Dimension: 3000 μm × 1500 μm × 22 μm	570 and 575 Hz	1.127 Vp-p, 0.123 μW	[49]
PZT-based energy harvester	The device is packed with the help of two wafers	1.8 kHz	40 μW	[52]
Thick film PZT cantilever beam to operate in d31 mode	Dimension: 13.5 mm × 9 mm × 192 μm	229 Hz	270 nW at 9.81 m/s^2^; 130 V	[53]
Two-layer PMNZT microgenerator	Dimension: 10 mm × 10 mm	120 Hz	2.0 Vp-p 0.5 mW	[54]
Piezoelectric cantilever/Laser machined	10 cantilevers with dimensions of 5.74 mm × 4 mm, 5 had tip masses attached	870 Hz	1.13 μW at 870 Hz through 288.5 kΩ, power density of 301.3 μW/cm^3^	[55]

## Data Availability

Not applicable.
